# Immunophenotypic Landscape of synovial tissue in rheumatoid arthritis: Insights from ACPA status

**DOI:** 10.1016/j.heliyon.2024.e34088

**Published:** 2024-07-04

**Authors:** JianBin Li, PengCheng Liu, YiPing Huang, Yan Wang, Jun Zhao, ZhenFang Xiong, MengXia Liu, Rui Wu

**Affiliations:** a Department of Rheumatology and Immunology, The First Affiliated Hospital, Jiangxi Medical College, Nanchang University, Nanchang, 330006, China; b Department of Pathology, The First Affiliated Hospital, Jiangxi Medical College, Nanchang University, Nanchang, 330006, China

**Keywords:** Circulating anti-citrullinated protein antibodies, Synovial tissue histopathological examination, Rheumatoid arthritis

## Abstract

**Objective:**

To examine the clinical features and synovial pathologies in rheumatoid arthritis (RA) patients across varying titers of circulating anti-citrullinated protein antibodies (ACPA).

**Methodology:**

We devised a negative pressure suction and rebound synovial biopsy tool to enhance the yield of synovial biopsies, noted for its ease and safety of use. This research involved a retrospective examination of 60 active RA patients who underwent synovial biopsies with this tool from June to November 2023 at our institution. A range of disease activity markers were collected, including DAS28-CRP, ESR, CRP, count of swollen and tender joints, VAS pain scale, and so forth. Synovial tissue underwent HE staining and immunohistochemistry, including synovitis grading (GSS) and counting of B cells (CD20), T cells (CD3), macrophages (CD68), and plasma cells (CD138).

**Participants:**

were categorized into three groups as per ACPA titers: ACPA-negative (0–5U/mL), low-titer (5–20U/mL), and high-titer (above 20U/mL). The study compared the clinical features and synovial pathologies across these groups.

**Results:**

Of the 60 RA patients, they were segregated into three groups based on ACPA titers: 20 in ACPA-negative, 9 in the low-titer group, and 31 in the high-titer group. No significant differences were observed in GSS scores, synovial cell proliferation and loss, matrix activation, inflammatory infiltration, and neovascularization among these groups (P > 0.05). The high-titer ACPA group demonstrated significantly increased counts of CD3^+^ T cells, CD20^+^ B cells, and CD68^+^ macrophages in synovial tissues compared to the ACPA-negative and low-titer groups (p < 0.05), along with a higher incidence of ectopic lymphoid neogenesis (p < 0.05). Ordinal logistic regression revealed that rheumatoid factor (RF), and counts of synovial T cells, B cells, macrophages, and ectopic lymphoid neogenesis correlated with ACPA titers (P < 0.05), particularly lymphoid neogenesis (OR = 3.63, P = 0.023).

**Conclusion:**

RA patients with high-titer ACPA demonstrate elevated levels of inflammatory cell infiltration in synovial tissues, with ectopic lymphoid neogenesis showing a strong correlation with high ACPA positivity.

## 1 Introduction

Rheumatoid arthritis (RA) is a chronic autoimmune disease that imposes a significant burden on patients, with its high disability rate and widespread prevalence affecting approximately 1 % of the global population [1]. Anti-citrullinated protein antibodies (ACPA) are widely used for the diagnosis of RA and are closely associated with joint damage and treatment response in patients [2]. Studies classify RA patients based on the presence or absence of anti-citrullinated protein antibodies, revealing different risk factors, pathogenesis, and treatment strategies for different ACPA statuses [3,4]. The presence of ACPA indicates a clinical subtype of RA, which often manifests as a more chronic, early joint erosion, a more destructive course of the disease, and more extra-articular symptoms [5].

In recent years, with the advancement of synovial biopsy techniques, research on RA synovial tissue has expanded. Studies have confirmed that ACPA concentrations in the synovium of RA are much higher than those in the serum [6], and there is an accumulation of B cells and plasma cells in the synovium [7]. Among them, a specific clone of plasma cells secreting ACPA has been identified, associated with the maturation of antigen-driven B cells and the production of antibodies in the synovium [8]. The presence of B cells and the ACPA-positive status may serve as predictive indicators for the chronicity of synovial inflammation and the responsiveness to B cell therapy [9]. Although there is evidence that ACPA plays a direct role in the pathology of RA synovitis, the specific immunological and inflammatory characteristics of synovium, as well as the mechanisms, remain unclear [10]. Therefore, this study aims to compare the clinical characteristics and synovial tissue histopathological examination of RA patients with different ACPA titers, further elucidating the pathological role of ACPA in RA synovitis and the predictive role of RA synovial features in disease progression and prognosis.

## 2 Methods

### 2.1 Patients

A retrospective study was carried out on RA patients who received synovial biopsies in the Department of Rheumatology and Immunology at the First Affiliated Hospital of Nanchang University between June and November 2023.Every patient fulfilled the criteria set by the American College of Rheumatology/European League Against Rheumatism (ACR/EULAR) in 2010 [11].All patients underwent synovial tissue sampling from joints showing active inflammation. Written informed consent was secured from each participant. The ethical review board of the First Affiliated Hospital of Nanchang University granted ethical approval for this study under the ethics number: IIT[2023] Clinical Ethics Review No. 011.Data on patients, including age, gender, disease duration, and medication history, were collected. Clinical data at the time of synovial biopsy, such as disease activity scores (DAS28-CRP, ESR, CRP), pain VAS scores, joint function evaluations, and autoantibody levels including RF and ACPA, were also gathered.Biopsy samples of synovial tissue were taken from inflamed joints, comprising 46 knee joints (76.67 %), 8 wrist joints (13.33 %), 2 elbow joints (3.17 %), and 4 ankle joints (6.35 %).

### 2.2 Synovial biopsy, staining, and tissue processing

**Fig. 1:**
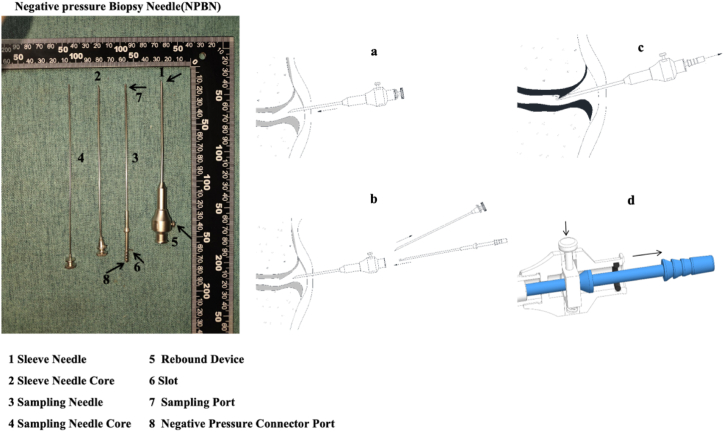
A blind Percutaneous Aspiration cutting synovial biopsy needle and its Working Principle Schematic Diagram.

Synovial tissue samples were obtained from affected joints using minimally invasive procedures with an improved Parker Pearson biopsy needle. The biopsy needle was modified to include a negative pressure device and a rebound mechanism. After the sheath needle entered the joint cavity, the core needle was removed, and the sampling needle was inserted. After connecting the sampling needle to the negative pressure device and creating a significant negative pressure, the rebound switch was pressed. The sampling needle rapidly rebounded, effectively cutting and obtaining synovial tissue (as shown in Fig. 1). Each sampling procedure yielded 6–7 fragmented tissue pieces, each measuring approximately 2 × 3mm.

The collected tissue samples were subjected to cryosectioning for hematoxylin and eosin (HE) staining and immunohistochemistry. Histological examination was conducted to assess tissue inflammation and structural changes, including synovial cell proliferation, detachment, matrix activation, neovascularization, and inflammatory cell infiltration. Each aspect was semi-quantitatively scored from 0 to 3. The Global Synovitis Score (GSS) was utilized to evaluate synovitis, with a score exceeding 4 supporting the diagnosis of inflammatory arthritis [12].

Immunohistochemistry was employed to examine the presence of immune cells expressing lineage markers (CD3, CD68, CD20, and CD138). Paraffin sections were deparaffinized, dehydrated, subjected to microwave heating, and underwent antigen retrieval in 10 mM citrate buffer (pH 6.0) for 15 min. Endogenous peroxidase activity was quenched with 3 % H2O2. Sections were then incubated overnight at 4 °C with primary antibodies, followed by incubation with corresponding secondary antibodies. Slides were developed using 3,3′-diaminobenzidine, counterstained with hematoxylin, and finally mounted in a non-aqueous mounting medium. Negative control slides (without primary antibody incubation) were included in each staining run.

The presence of lymphocyte aggregates was assessed on anti-CD3–stained sections, and aggregates were counted. Two senior pathologists, blinded to the relevant clinical data, independently scored the intimal and subintimal layers of the synovium. Differences in scores between observers were resolved through joint review, reaching a consensus in each case.

### 2.3 ACPA test by CIA

Blood samples (5 mL) were collected from fasting study participants in the morning, centrifuged at 3000r/min for 10 min, with the serum subsequently separated and stored at −80 °C for future analysis.Serum levels of ACPA were determined using an automated chemiluminescence method. targeting the CCP2 antigen [13].The assay kit was sourced from Yuhuilong Company, and the YQMY218 fully automated chemiluminescence analyzer from Yuhuilong Biotechnology Co., Ltd. was employed, setting the ACPA threshold at 5 units.Procedures were meticulously followed as per the instructions provided with the kit and the device manual. Participants were categorized into three groups according to ACPA titers: ACPA + high titer group (ACPA >20U/mL), ACPA + low titer group (5U/mL ≤ ACPA ≤20U/mL), and ACPA-negative (<5U/mL).

### 2.4 Statistics

Statistical analysis was performed using SPSS 26.0.The Kolmogorov-Smirnov test was used to assess the normality of the data.For normally distributed continuous and ordinal data, mean and standard deviation (SD) were used for representation, while non-normally distributed data were represented by the median and interquartile range (IQR).Group differences in normally distributed data were assessed using unpaired (two-tailed) t-tests.For non-normally distributed data, group differences were analyzed using the Mann-Whitney *U* test.Categorical data were represented as the number and percentage of participants, with group differences analyzed using either Fisher's exact test or chi-square test.Ordinal logistic regression analysis was employed to screen for indicators related to ACPA titers, with a P-value below 0.05 deemed statistically significant.

## 3 Results

### 3.1 Patients and Outcomes

This study involved 60 patients with rheumatoid arthritis, whose baseline demographic and disease characteristics are presented in Table 1.The positivity rate for ACPA was 66.67 %. Patients were categorized into three groups according to ACPA titers: ACPA + high titer group, ACPA + low titer group, and ACPA-negative.Except for RF, which showed a significant difference (P < 0.05), there were no significant differences in other factors such as age, disease duration, medication, ESR, CRP, DAS28, etc., among the three groups.However, RA patients who were ACPA + did have higher median counts of swollen and tender joints and higher DAS 28 scores (as indicated in Table 1).

**Table 1:** Baseline demographics and clinical features.

	ACPA-negative	low titer group	high titer group	P
n	20	9	31	
Female(%)	19(95)	9(100)	27(87.10)	0.147
Age,years	50.55 ± 13.22	54.44 ± 12.52	55.87 ± 9.36	0.261
Rheumatoid factor (iu/ML)	19.70(0–58.74)	118.02(49.75–248.09)	141.57(47.10–333.64)	0.003
Disease Duration, months	54(8.25–165)	120(78–180)	72(24–120)	0.308
DAS28 CRP	4.55 ± 1.00	4.74 ± 1.01	4.81 ± 1.06	0.669
CRP mg/L	21.78(6.31–73.96)	17.92(9.43–33.99)	24.99(6.51–66.7)	0.914
ESR mm/Hr	32.5(20.25–51.25)	49(28–71)	45(17–67)	0.548
SJC 28	4(1–6)	5(1–7)	5(2–8)	0.413
TJC 28	4(2–6)	6(2.5–9)	5(2–8)	0.400
VAS	60(60–70)	60(50–75)	70(60–80)	0.424
Treatment,n(%)				
Methotrexate	10(50)	5(55.56)	19(61.29)	0.723
JAKi	2(10)	2(22.22)	2(6.45)	0.301
TNF-a inhibitor	5(25)	4(44.44)	5(16.13)	0.099
IL-6 inhibitor	1(5)	1(11.11)	2(6.45)	0.730

### 3.2 **Synovial characteristics by hematoxylin-eosin** staining **in different ACPA status groups**

Between the three groups, there were no significant differences in GSS scores, synovial cell loss, synovial cell proliferation, matrix activation, inflammatory cell infiltration, or neovascularization (P > 0.05) (Table 2).

**Table 2:** Characteristics of synovial tissue (HE staining) in the three ACPA groups of RA patients.

	ACPA-negative	low titer group	high titer group	P
Global Synovitis Score	3(2–5.25)	4(1.5–4.5)	4(3–5)	0.828
Synovial hyperplasia grade,n(%)				0.598
0	7(35)	3(33.3)	13(41.9)	
I	5(25)	5(55.6)	10(32.2)	
II	1(5)	1(11.1)	5(16.1)	
III	1(5)	0	0	
Detachment,n(%)	2(10)	0	6(19.35)	0.302
Stromal activity grade,n(%)				0.599
0	0	1(11.1)	2(6.5)	
I	9(45)	7(77.8)	18(58.1)	
II	5(25)	1(11.1)	8(25.8)	
inflammatory score grade,n(%)				0.656
0	1(5)	1(11.1)	1(3.2)	
I	7(35)	3(33.3)	7(22.6)	
II	4(20)	4(44.4)	16(51.6)	
III	2(10)	1(11.1)	3(9.7)	
neovascularization grade,n(%)				0.219
0	0	0	2(6.5)	
I	3(15)	5(55.6)	10(32.3)	
II	6(30)	3(33.3)	4(12.9)	
III	5(25)	1(11.1)	11(35.5)	

### 3.3 Immune cells in different ACPA status

**Fig. 2:**
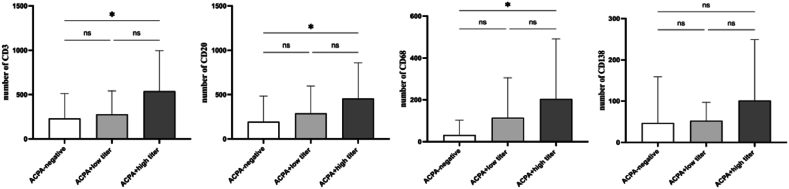
Cell counts of CD3, CD20, CD68, and CD138 in three groups of RA patients based on ACPA status.Statistical significance is indicated by p < 0.05.*.

**Fig. 3:**
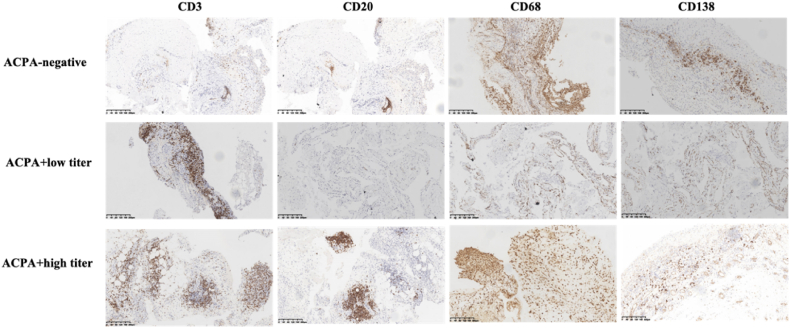
Immunohistochemical images of synovial tissue illustrating CD3, CD20, CD68 and CD138 labellings in the three ACPA groups.Original mag 20x.

According to Table 3 and Fig. 2, the synovium of the ACPA + high titer group displayed increased expression of CD3^+^ T cells, CD20^+^ B cells, and CD68^+^ macrophages (P < 0.05).Fig. 3 illustrates the distribution of immune cells, including CD3, CD20, CD68, and CD138, in the synovium of three representative patient groups, as demonstrated by immunohistochemical staining.

**Table 3:** Immune Cell Infiltration in Three groups of RA patients based on ACPA status.

	ACPA-negative	low titer group	high titer group	P
CD3,n	232 ± 280.46	277.78 ± 266.12	540 ± 456.47	0.016
CD20,n	194.25 ± 288.09	288.89 ± 309.01	455.33 ± 402.78	0.042
CD68,n	32.50 ± 70.03	114.44 ± 191.64	199 ± 284.17	0.039
CD138,n	47 ± 112.07	52.22 ± 44.66	101.29 ± 147.87	0.279

### 3.4 Lymphoid neogenesis in different ACPA status groups

**Fig. 4:**
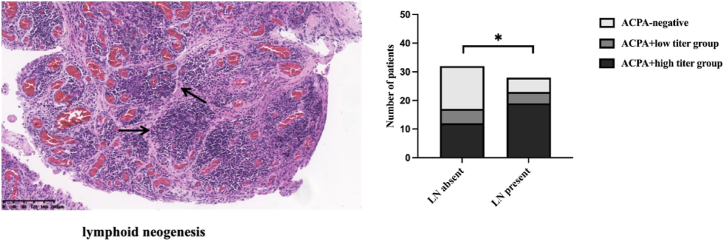
Representative Images of Lymphoid Neogenesis and Differential Presence Across ACPA Titers.Statistical significance is indicated by p < 0.05.* Original mag 10x.

Within the ACPA + high titer group, 61.29 % of patients had lymphoid neogenesis (as shown in Fig. 4), compared to 44.44 % in the ACPA + low titer group and 25 % in the ACPA-negative group.Chi-square analysis (Fig. 4) revealed significant differences in the distribution of lymphoid neogenesis among the synovial tissues of the three groups (P < 0.05).

### 3.5 Analysis **of the correlation between ACPA titers and clinical characteristics and synovial tissue histopathological examination**

CD3 (T lymphocyte count < or ≥1000), CD20 (B lymphocyte count < or ≥800), and CD68 (macrophage count < or ≥50) were divided into two groups each. In the univariate logistic analysis, grouping of CD3, CD20, CD68, and lymphoid neogenesis showed significant correlation with ACPA titer categories (P < 0.05).In the multivariate analysis, factors such as disease duration, age, and medication were adjusted for as confounders. In the revised model, RF (OR 1.01, 95 % CI 0.001–0.009, P = 0.025), CD3 (OR 5.88, 95 % CI 0.430–3.014, P = 0.009), CD20 (OR 3.63, 95 % CI -0.005-0.007, P = 0.049), CD68 (OR 3.63, 95 % CI 0.177–2.405, P = 0.0023), and lymphoid neogenesis (OR 4.26, 95 % CI 0.419–2.580, P = 0.007) were still significantly relevant (Table 4).

**Table 4:** Ordinal logistic regression model showing the correlation of different indicators with ACPA titers.

	Univariate		Multivariate	
	OR(95CI)	P value	OR(95CI)	P value
CD3	4.94(0.335–2.860)	0.013	5.88(0.430–3.014)	0.009
CD20	3.61(0.012–2.556)	0.048	3.63(-0.005-0.007)	0.049
CD68	3.42(0.148–2.317)	0.026	3.63(0.177–2.405)	0.023
lymphoid neogenesis	3.70(0.279–2.336)	0.013	4.26(0.419–2.580)	0.007

## 4 Discussion

Autoantibodies and immune complexes have been recognized as potent pathological triggers of inflammation for a long time.A reasonable explanation for synovial tissue damage in RA is the local deposition of immune complexes formed by autoantibodies.RF and ACPA are the two most prevalent autoantibodies in RA. RF, which specifically targets the constant region of IgG, is detectable in over 80 % of RA patients [14].It is reported that IgM RF comprises more than 10 % of plasma cells in RA synovium [15,16]. Infusing RF into healthy individuals neither induces persistent nor transient synovitis, suggesting that RF autoantibodies are not inherently pathogenic [17].The positivity rate of ACPA in early RA is approximately 50 % [18]. In this study, the positivity rates for RF and ACPA were 83.33 % and 66.67 %, respectively.Relative to RF, ACPA plays a more direct role in RA, especially in the aspect of joint destruction [19].The production of ACPA results from the immune system's aberrant response to specific peptides, leading to chronic inflammation, tissue damage [20], bone erosion, and cartilage destruction [21].Research indicates that ACPA can intensify inflammation by activating certain immune cells and releasing pro-inflammatory cytokines like tumor necrosis factor α and interleukin-6 [22,23], hence ACPA-positive RA may display greater disease activity and more severe joint structural damage than those who are ACPA-negative [24].

RA synovium usually displays chronic inflammation, characterized by the infiltration of numerous immune-inflammatory cells, predominantly lymphocytes, plasma cells, and macrophages.While no differences were noted in the semi-quantitative scoring of inflammatory cell infiltration in the synovium GSS among RA patients with varying ACPA titers (P > 0.05), immunohistochemistry indicated a significant elevation in certain immune cells at higher ACPA titers.Previous research has shown significant differences in synovitis between ACPA-positive and ACPA-negative RA patients, especially in lymphocyte infiltration. ACPA-positive RA patients often exhibit a faster rate of local joint destruction [25]. Consistent with these findings, our study observed that in the RA synovial tissues of the high ACPA titer group, the numbers of CD20^+^ B cells and CD3^+^ T cells were significantly higher than those in the low ACPA titer group and ACPA-negative RA patients. Additionally, a prospective study indicated that during follow-up, arthritis occurred in 15 (27 %) RA patients who were ACPA or RF positive. Although significant synovitis was not observed in the biopsy tissues, the number of CD3^+^ T cells showed a critical positive correlation with the subsequent development of clinical arthritis in these patients [26]. Another prospective study further confirmed that the levels of CD3^+^ T cells in the synovium of ACPA-positive RA patients significantly increased, and the extent of synovial B cell infiltration and lymphocyte aggregation in these patients was significantly higher, especially in the untreated patient population [27]. Such differences lead to more severe forms and sustained inflammatory progression in the ACPA-positive subtype.Additionally, this study further substantiates through multifactorial logistic regression analysis that the infiltration levels of CD3^+^ T lymphocytes and CD20^+^ B lymphocytes significantly correlate with ACPA titers (OR = 5.88, 3.63; P = 0.009, 0.049).T and B lymphocytes are significant in the context of ACPA-positive RA [3].T cells, being the most prevalent immune cells in RA synovitis, play a dual role. Their infiltration initiates and sustains the activation of macrophages and synovial fibroblasts, converting them into tissue-destructive effector cells, and they also provide the essential second signal for B cell activation, crucial for ACPA production.Elevated ACPA levels can precede joint symptoms and collaboratively work with shared HLA-DR antigen epitopes, heightening the risk for RA development [28].Besides autoantibody production, B cells also efficiently present antigens to T cells, produce soluble mediators such as cytokines and chemokines, and form aggregates in the target organs of rheumatoid arthritis.

Lymphocyte infiltration in the RA synovium can exhibit a diffuse distribution, accompanied by the formation of complex lymphoid microstructures, and induce Germinal Center (GC) reactions [29].These microstructures share many features with secondary lymphoid tissues, thus, the formation of GCs at extranodal sites can be regarded as lymphoid neogenesis.Studies have found evident aggregation of T cells and B cells in the synovial tissues of all RA patients, with approximately half of the synovial tissues exhibiting germinal center reactions [30].Another study indicated that 49 % of RA synovial tissues in 86 RA patients exhibited lymphoid neogenesis, and patients with lymphoid neogenesis had a significantly longer disease course [31].RA is one of only a few diseases in which ectopic germinal centre-like structures can be observed at the site of inflammation. These structures, which range from loose aggregates of T and B cells to distinct follicle-like structures, are often observed in close contact with the inflamed synovial membrane of RA patients.The effect of these lymphatic tissue structures on autoantibody production remains largely unknown.Research has discovered a link between circulating RF and both the presence [32] and eradication [33] of Germinal Center reactions in the synovium.However, there are also studies that refute this association [34].RA synovia containing lymphoid aggregates have significantly larger amounts of RF-IgM and anti-CCP IgG, after normalizing for serum content [35], and lymphoid aggregates may provide an important environment for B cells producing autoantibodies related to arthritis, allowing precursor B cells to proliferate and undergo highly variable hypermutation, then differentiate into (auto)antibody-producing plasma cells [36].CD3+ T cells also participate in the formation of ectopic germinal centers in RA synovial tissues, and these germinal centers play a crucial role in antibody formation [37].Therefore, the existence of lymphoid neogenesis likely contributes to the production of ACPA and RF.

Our research additionally discovered a notable rise in CD68^+^ macrophages in ACPA-positive RA patients (both high titer and low titer groups). Logistic regression analysis indicated a significant association between the infiltration of CD68^+^ macrophages and ACPA titers (OR = 3.63, P < 0.05), an association not previously documented.Synovial Tissue Macrophages are situated across the sub-lining and lining layers at the cartilage-pannus junction, playing a role in joint destruction.Sub-lining macrophages are currently viewed as the most dependable biomarker for determining disease severity and response to treatment in RA.STMs play a pro-inflammatory role in RA, being the main producers of the pathogenic tumor necrosis factor (TNF).Research has shown that suppressing the expression of macrophages in synovium can effectively reduce arthritis in RA model rats [38].Studies have discovered that ACPAs are deposited at the citrulline sites in CD68 positive cells of RA synovium [39], potentially contributing to enhanced activation and infiltration of STMs and increasing the erosiveness of ACPA-positive synovium.

This research, being a single-center retrospective study, has a comparatively small sample size and did not uncover correlations of other RA synovitis characteristics, like synovial cell proliferation, matrix cell activation, and angiogenesis, with ACPA titers. Future larger, prospective studies are essential to further validate these findings and investigate the underlying mechanisms.

## 5 Conclusion

RA patients with high titers of ACPA show increased infiltration of T, B, and macrophage cells, as well as ectopic lymphoid proliferation in synovial tissues. This discovery holds substantial importance for understanding RA's pathogenesis and devising more effective treatments.

## Funding

The National Natural Science Fund of China: 82260898.

## Data availability

Primary data are available upon the corresponding author request.

## Ethics approval and consent to participate

The ethical review board of the First Affiliated Hospital of Nanchang University granted ethical approval for this study under the ethics number: IIT[2023] Clinical Ethics Review No. 011.

## Consent for publication

Not applicable.

## CRediT authorship contribution statement

**JianBin Li:** Writing – original draft, Data curation. **PengCheng Liu:** Software, Data curation. **YiPing Huang:** Visualization, Data curation. **Yan Wang:** Conceptualization. **Jun Zhao:** Methodology. **ZhenFang Xiong:** Validation, Supervision. **MengXia Liu:** Data curation. **Rui Wu:** Writing – review & editing.

## Declaration of competing interest

The authors declare that they have no known competing financial interests or personal relationships that could have appeared to influence the work reported in this paper.

## Abbreviations

RA: Rheumatoid arthritis
ACR: American College of Rheumatology
EULAR: European League Against Rheumatism
RF: Rheumatoid factor
DAS28: Disease Activity Score in 28 Joints
JAKi: JAK inhibitors
ESR: Erythrocyte sedimentation rate
CRP: C-reactive protein
ACPA: Anti-citrullinated protein antibodies
HE: Hematoxylin and eosin
GSS: Global Synovitis Score
GC: Germinal Center
TNF: Tumor necrosis factor
LN: lymphoid neogenesis
